# CD73 Expression on Mesenchymal Stem Cells Dictates the Reparative Properties via Its Anti-Inflammatory Activity

**DOI:** 10.1155/2019/8717694

**Published:** 2019-05-02

**Authors:** Kezhe Tan, Hongtao Zhu, Jianfang Zhang, Weili Ouyang, Jianfeng Tang, Youming Zhang, Linlin Qiu, Xueqing Liu, Zhaoping Ding, Xiaoming Deng

**Affiliations:** ^1^Department of Anesthesiology, Changhai Hospital, Naval Medical University, Changhai Rd. 168, 200433 Shanghai, China; ^2^Department of Cardiology, Danyang People's Hospital, West Xinmin Rd. 5, 212300 Danyang, China; ^3^Institute of Molecular Cardiology, Heinrich-Heine University of Düsseldorf, Moorenstr. 5, 40225 Düsseldorf, Germany

## Abstract

Mesenchymal stem cells (MSC) are not universal and may be subject to dynamic changes upon local milieus in vivo and after isolation and cultivation in vitro. Here, we demonstrate that MSC derived from murine pericardial adipose tissue (pMSC) constitute two cohorts of population distinguished by the level of CD73 expression (termed as CD73^high^ and CD73^low^ pMSC). Transplantation of two types of cells into mouse hearts after myocardial infarction (MI) revealed that the CD73^high^ pMSC preferentially brought about structural and functional repair in comparison to the PBS control and CD73^low^ pMSC. Furthermore, the CD73^high^ pMSC displayed a pronounced anti-inflammatory activity by attenuating CCR2^+^ macrophage infiltration and upregulating several anti-inflammatory genes 5 days after in vivo transplantation and ex vivo cocultivation with peritoneal macrophages. The immunomodulatory effect was not seen in cocultivation experiments with pMSC derived from CD73 knockout mice (CD73^−/−^) but was partially blocked by pretreatment of the A2b receptor antagonist, PSB603. The results highlight a heterogeneity of the CD73 expression that may be related to its catalytic products on the modulation of the local immune response and thus provide a possible explanation to the inconsistency of the regenerative results when different sources of donor cells were used in stem cell-based therapy.

## 1. Introduction

Myocardial infarction (MI), commonly known as heart attack, results in the loss of around 1 billion heart muscle cells and the destruction of surrounding blood vessels. Once damaged, adult heart cells cannot be replaced through their own regeneration; therefore, an alternate form of wound healing is orchestrated by the immune system [1, 2]. Inflammatory cells migrate to the injured heart, ensuring the clearance of harmful cell debris and repair of the damaged area via the formation of a fibrotic scar [3]. While the default immune response to MI appears to ensure a quick fix of the heart, the scarring leads to pathological remodeling of the heart and compromised cardiac function over time. Moreover, if the initial acute inflammatory response becomes chronic, host tissue will be subject to continued damage [4].

There is a growing body of evidence supporting the hypothesis that stem cell-based therapy may work via paracrine-mediated regulation on the local immune response in the damaged heart [5]. Mesenchymal stem cells (MSC) are able to produce and secrete a broad variety of cytokines, chemokines, and growth factors that may potentially modulate excessive inflammatory response that, following MI, constitutes a proinflammatory milieu, much of which is cytotoxic, and an ensuing fibrosis that disrupts the essential matrix [6]. Therefore, the immunoregulatory property of MSC by means of either paracrine factors, exosmotic vesicles, or others is functionally linked to their reparative activity [5, 7] and determines the therapeutic potential in vivo [8]. However, the variability in terms of immunomodulation among MSC from different anatomic origins and biological properties is often ignored, as MSC are broadly assumed with similar epitope identities and differentiation potentials, which implies that multiple tissues are equally suitable cell sources for the regeneration of multiple tissues [9].

Indeed, the MSC pool is a rather heterogeneous population that varies dynamically in response to local milieus [10] and may be subject to changes in biological functionality after in vitro isolation and expansion [11]. Furthermore, isolated MSC even from the same source display a heterogeneous transcriptional profile that links to cellular communication in the heart [12]. Notably, MSC isolated from different murine tissues showed a diverse capacity to metabolize extracellular nucleotides, a process which requires the presence of ecto-5′-nucleotidase activity (eNT/CD73) [13]. Given that CD73 is one of the classic markers that specifically define the MSC population, we therefore hypothesize that the nonuniform expression of CD73 on MSC may be associated with the reparative property, as extracellular adenosine catalyzed by the dephosphorylation activity of CD73 has been shown as a pivotal regulator of local immune response [14].

In the present study, we demonstrate that MSC derived from pericardial adipose tissue [15], termed as pMSC, bear the heterogeneous expression of CD73 and that the CD73-enriched pMSC favors cardiac repair via anti-inflammatory activity.

## 2. Materials and Methods

### 2.1. Animal Experiments

All animal experiments were approved by the Experimental Animal Care and Use Committee at the Navy Medical University and were performed in accordance with institutional guidelines on animal care. A total of 52 mice (48 × C57BL/6wild type and 6 × CD73^−/−^ with body weight of 20–25 g and age of 8–12 weeks and 6 × Wistarrats) were bred at the central animal facilities of the Navy Medical University and fed with a standard chow diet/tap water ad libitum.

A murine model of myocardial infarction (MI, 60 min ischemia+reperfusion) was induced as previously described [16]. In brief, mice were intubated and anesthetized by mechanical ventilation with isoflurane (1.5% *v*/*v*) in 80% oxygen/20% nitrogen. The animal was placed in a supine position, and the chest was opened with a lateral cut along the left side of the sternum. The left anterior descending coronary artery (LAD) was visualized with a microscope and ligated by a suture (8-0 polypropylene) with a soft, home-made vessel protecting the pad above the LAD. The success of occlusion was assured by ST-segment elevation in ECG recordings and the presence of myocardial blanching in the perfusion bed. Ligation was maintained for as long as 60 min to fulfill a transmural MI until the suture was released to allow reperfusion. In the cell transplantation groups, mice intramyocardially received 20 *μ*l of suspension in PBS containing 2 × 10^5^ of either CD73^low^ or CD73^high^ pMSC at two separate sites within the infarct area using an insulin syringe (30 G, BD) shortly after releasing the ligation. The chest was then closed with one layer through the muscle and a second layer through the skin, and the animals were then weaned from ventilation and placed in a warm and oxygen-enriched environment until they fully recovered.

The animals were ready for sacrifice at two time points: 5 days and 28 days after the surgical procedure. The 5-day animals were used for the analysis of the inflammatory statue of the hearts and the 28-day animals for structural and functional repair after cardiac damage (MI).

Cardiac function was assessed by transthoracic echocardiography using a high-frequency, high-resolution digital imaging platform for small animals with an 18 to 38 MHz transducer MS400 (Vevo 2100; VisualSonics, Canada). Briefly, the animals were anesthetized by inhalation of isoflurane (1.5% *v*/*v*) using a home-made mask, and the short axes at the midventricular sections were acquired in M-mode. The data were stored, and parameters of cardiac function were analyzed off-line with customized software (VisualSonics).

After the measurements of functional parameters, the animals were sacrificed with CO_2_ and the hearts were excised. After being washed once in ice-cold PBS, the hearts were carefully positioned and embedded in a Tissue-Tek® (Leica, Germany) for histological analysis.

### 2.2. Isolation of Mesenchymal Stem Cells from Various Tissues

Pericardial and subcutaneous adipose mesenchymal stem cells (MSC) were isolated according to the modified protocol previously reported [10]. In brief, either C57BL/6 wild-type (WT) or CD73 knockout (CD73^−/−^) mice were killed using CO_2_ suffocation. Under sterilized conditions, the chest was opened and the subcutaneous and pericardial adipose tissue was excised. The collected pericardial tissue was then cut into small pieces and transferred into a 2 ml digestion solution containing 0.4% collagenase II (Biochrom, Germany) and incubated at 37°C with gentle rotation (20 rpm) for 20 minutes before digestion was stopped by the addition of 2 ml of FCS (HyClone, USA). The resultant cell suspension was spun down at 1,460 rpm for 5 minutes, and the cell pellet was resuspended with basic medium containing low-glucose DMEM and supplemented with 30% FCS, penicillin (100 U/ml), streptomycin (0.1 mg/ml), and glutamine (2 mM). The isolated cells were inoculated into a 10 cm petri dish and incubated at 37°C with 5% CO_2_. The adherent stromal vascular fraction was then termed as pMSC. Cell numbers were counted in a small aliquot, and the population-doubling time was calculated.

Bone marrow MSC (bMSC) were obtained from mice and umbilical cord blood MSC (ucMSC) from humans [17]. In brief, ucMSC were isolated as mononuclear cells by Ficoll gradient centrifugation from human donors, and bMSC was obtained from mouse femurs and tibiae flushed with DMEM medium. The cell suspension was both filtered and plated into a 6-well plastic cell culture plate at a density of 2 × 10^5^ cells and cultured in a similar condition as mentioned above for the analysis of the CD73 expression profile.

**Figure 1:**
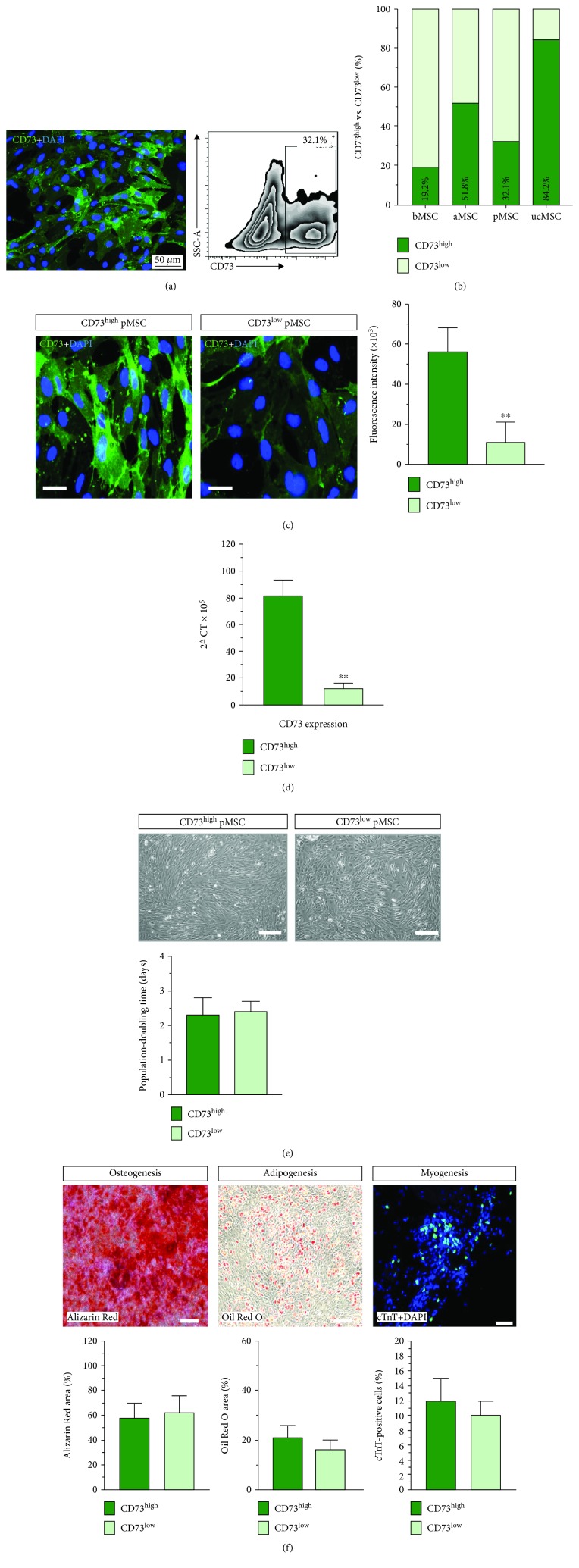
Nonuniform CD73 expression in the MSC pool. Two subsets of isolated pMSC were found upon the intensity of the CD73 expression, nearly one-third of the population with a high expression of CD73 and the rest with a low expression (a). Nonuniform aspect of the CD73 expression was also found in MSC from the bone marrow (bMSC), subcutaneous adipose (aMSC), and human umbilical cord blood (ucMSC) (b). By using FACS separation, expression of CD73 at a high level (CD73^high^ pMSC) and expression of CD73 at a low level (CD73^low^ pMSC) constitute a fivefold difference according to fluorescence intensity (c) and eightfold at the mRNA level (d). Both subsets of pMSC showed a typical cobblestone morphology and had similar population-doubling time (e) and identical potential in osteogenic, adipogenic, and myogenic differentiation (f). Bar = 50 *μ*m in (a), 20 *μ*m in (c), and 100 *μ*m in (e) and (f). ^∗∗^ indicates *p* < 0.01.

To separate two subpopulations of pMSC that constitute different CD73 expressions, the cultivated cells (passage 1) were gently detached with 0.05% trypsin EDTA (Sigma-Aldrich). After washing once with cold PBS, about 1 × 10^5^ cells per 100 *μ*l suspension were incubated with Alexa Fluor® 488- (AF488-) conjugated antibody against CD73 (1 : 100, BD Pharmingen) at 4°C void of light for 10 min. Thereafter, the unbound antibody was removed by washing twice with 2 ml MACS buffer, and the cells were separated according to the intensity of CD73 staining (fluorescence) with the help of the Core Flow Cytometry Facility at the Naval Medical University using fluorescence-activated cell sorting (FACS, Moflo XDP, Beckman Coulter) (Figure 1(a)). As such, according to the expression intensity of CD73, two pMSC populations, namely, the CD73 high expression (CD73^high^) and the CD73 low expression (CD73^low^), were subdivided. Sorted pMSC were cultured with 30% FCS low-glucose DMEM and the other components mentioned above at 37°C 5% CO_2_ for further experiments.

### 2.3. Induction of Myogenic, Adipogenic, and Osteogenic Differentiation

To compare differentiation potentials, both the CD73^low^ and CD73^high^ pMSC at passage 2 were seeded at a density of 2 × 10^4^ cells/cm^2^ in basal medium. Adipogenic induction was induced by a high glucose adipogenic induction medium (Gibco), supplemented with 10% FCS, 1% L-glut, 1% Pen-Strep, 1 *μ*M dexamethasone, 1 *μ*M indomethacin, 500 *μ*M 3-isobutyl-1-methylxanthine (IBMX), and 10 *μ*g/ml human recombinant insulin as previously reported [15]. Osteogenic differentiation was induced by osteogenic induction medium (Cyagen), containing 10% FCS, 1% Pen-Strep, 1 *μ*M dexamethasone, 50 *μ*M ascorbate, and 10 *μ*M *β*-glycerophosphate. Induction was maintained for 2 weeks before Oil Red O (adipogenesis) and Alizarin Red (osteogenesis) staining was performed.

For myogenic differentiation, both CD73^low^ and CD73^high^ pMSC were seeded at a density of 3.1 × 10^3^ cells/cm^2^ into eight-chamber slides (Nunc) and cultured in DMEM culture medium. As soon as subconfluence was reached, myogenic differentiation was induced by changing the culture medium into DMEM supplemented with only 5% FCS, 5% horse serum together with 0.1 mM of 5-azacytidine (Sigma-Aldrich), and 0.1 *μ*M of dexamethasone (Sigma-Aldrich) and verified by staining for cardiac troponin T (cTnT, Thermo Fisher Scientific). The percentage was derived by counting the number of cTnT-positive cells versus the total cell numbers (DAPI positive).

### 2.4. Isolation of Cardiac Immune Cells and Flow Cytometric Analysis

Five days after MI, mice were anaesthetized by intraperitoneal injection of pentobarbital (40-50 mg/kg), and the hearts were rapidly excised and mounted onto a Langendorff apparatus to establish a retrograde perfusion (perfusion pressure 100 mmHg, 37°C) for 5 min with an oxygenated medium (Krebs-Henseleit buffer: 116.02 mM NaCl, 4.63 mM KCl, 1.10 mM MgSO_4_·7H_2_O, 1.21 mM K_2_HPO_4_, 2.52 mM CaCl_2_·2H_2_O, 24.88 mM NaHCO_3_, 8.30 mM D-glucose, and 2.0 mM sodium pyruvate, containing 95% O_2_ and 5% CO_2_ infusion) before changing to a digestion solution containing collagenase II (1400 U/ml, Biochrom AG, Berlin, Germany) in phosphate-buffered saline for 25 min at 37°C. The collagenase-treated heart was removed from the cannula and weighed, and the atria were removed. The heart was then minced and pipetted as a single-cell suspension. The cell suspension was first meshed through a 100 *μ*m cell strainer (BD Falcon) and centrifuged at 55 g for one minute to separate cardiomyocytes from noncardiomyocytes. The supernatant containing the noncardiomyocyte fraction was again passed through a 40 *μ*m cell strainer (BD Falcon). The finally collected cells were resuspended in MACS buffer (2% FBS in PBS with 1 mM EDTA) and stained with multiple fluorescence-conjugated antibodies for flow cytometry (FACSCanto II; BD Biosciences) and analyzed by the FlowJo 7.6 software.

The following antibodies were used in the present experiment: AF488 or PE or APC-conjugated anti-CD73 (BD Pharmingen), PE-Cy7-conjugated anti-CD45 (BioLegend), APC-Cy7-conjugated anti-B220 (BD Pharmingen), APC-conjugated anti-CD11b (BD Pharmingen), FITC-conjugated anti-CD11c (BD Pharmingen), PE-conjugated anti-CD3 (BioLegend), PerCP-Cy5.5-conjugated anti-Ly6G (BD Pharmingen), APC-Cy7-conjugated anti-Ly6C (BD Pharmingen), and FITC-conjugated anti-CCR2 (BioLegend).

### 2.5. Cocultivation of pMSC with Peritoneal Macrophages

The rat peritoneal macrophage (pM*ϕ*) was used in the coculture experiments to ensure PCR detection. For isolation of pM*ϕ*, rats were intraperitoneally administered with 4% thioglycollate medium (20 ml) and peritoneal lavage was collected 3 days after injection. Cells were centrifuged down at 350 g for 5 minutes and resuspended with DMEM medium supplemented with 10% FCS, penicillin (100 U/ml), streptomycin (0.1 mg/ml), and glutamine (2 mM). After a 3-day cultivation on a petri dish, the attached cells were identified as pM*ϕ* by flow cytometry as CD11b^+^CD11c^−^ staining, indicating that more than 90% of the cells were CD11b^+^CD11c^−^ cells (data not shown). In respect to the coculture experiment, 0.4 *μ*m cell insertions (12-well plate sizes, Sigma-Aldrich) were applied and 5 × 10^5^ isolated pM*ϕ* were cultivated in each well of the 12-well plates. The coculture was achieved by addition of pMSC into the culture dish with the ratio of 1 : 2. In some experiments, pharmacological inhibition of A2b receptors by PSB603 (100 nM) was proceeded by 15 min of preincubation prior to the addition of pMSC. Cocultivation was maintained for 24 hours before the cells were collected for quantitative reverse transcription PCR (qRT-PCR).

### 2.6. Quantitative Real-Time RT-PCR

Real-time RT-PCR was performed to analyze the cytokine expression profile of the samples either from the heart tissue (5 days after MI) or from the pM*ϕ* after cocultivation. Total RNA was isolated from tissues and cells using the RNeasy Midi Kit (QIAGEN) and RNeasy Micro Kit (QIAGEN), and cDNA was synthesized using the QuantiTect Reverse Transcription Kit (QIAGEN) according to the manufacturer's instructions. All commercial primers (see below) were purchased from Thermo Fisher Scientific and worked properly, as indicated by the amplification plots. Gene analysis was conducted using the StepOnePlus Real-time PCR System following the manufacturer's protocol, and all PCR assays were performed in duplicate. Relative gene expression was normalized to GAPDH and calculated using the 2^Δ^ Ct value methodology. The following primers were used in the present experiments: CD73, Mm00501910_m1; NOS2, Rn00561646_m1; IL-4, Rn01456866_m1; Arg-1, Rn00691090_m1; TGM-2, Rn00571440_m1; IL-10, Rn01483988_g1; TNF-*α*, Rn01525860_g1; INF-*γ*, Rn00594078_m1; and GAPDH, Rn01775763_g1 (rat) and Mm99999915_g1 (mouse).

### 2.7. Cyto- and Histostaining

For cytostaining of CD73 on pMSC, freshly isolated pMSC were seeded into chamber slides for 4 days until 90% of cell confluency was reached. The cells were then washed and fixed with 1% paraformaldehyde for 10 min. After 1 h of blocking in 5% normal goat serum (NGS), cells were incubated for 2 hours with primary antibodies (polyclonal anti-CD73). After washing three times with 1% NGS-PBS buffer, secondary FITC-conjugated antibody (goat IgG, 1 : 100, Santa Cruz Biotechnology) was added and incubated for 60 min. Nuclei were counterstained with DAPI (DAKO), and the chamber slides were sealed with ProLong Gold® (Life Science).

For Sirius Red and HE staining, the heart samples embedded in Tissue-Tek were sliced into 10 *μ*m sections at the midventricular level. Slides in the midventricular section were stained with haematoxylin for 5 minutes, washed for 15 minutes with tap water, and stained with 1% eosin solution for an additional 1 minute. After several steps of dehydration with 75% to 100% ethanol, the slides could be covered with DPX mounting medium. All images were acquired and analyzed using light microscopy (Olympus BX51). The infarct size was derived from the percentage of collagen-stained ventricular wall in relation to the total circumference.

### 2.8. Statistical Analysis

Data are presented as mean ± standard deviation (SD). A Student *t*-test with Welch's correction was applied to compare the cytometric data cytokine expression profile treated with CD73^low^ and CD73^high^ pMSC. The structural (wall thickness) and functional (echo parameters) data and inhibitory data (PSB603) were compared with one-way analysis of variance (ANOVA). Differences were considered significant at *p* < 0.05. The Prism software package (version 7.0) was used for the statistical analyses.

## 3. Results

### 3.1. Nonuniform Expression of CD73 in MSC

**Figure 2:**
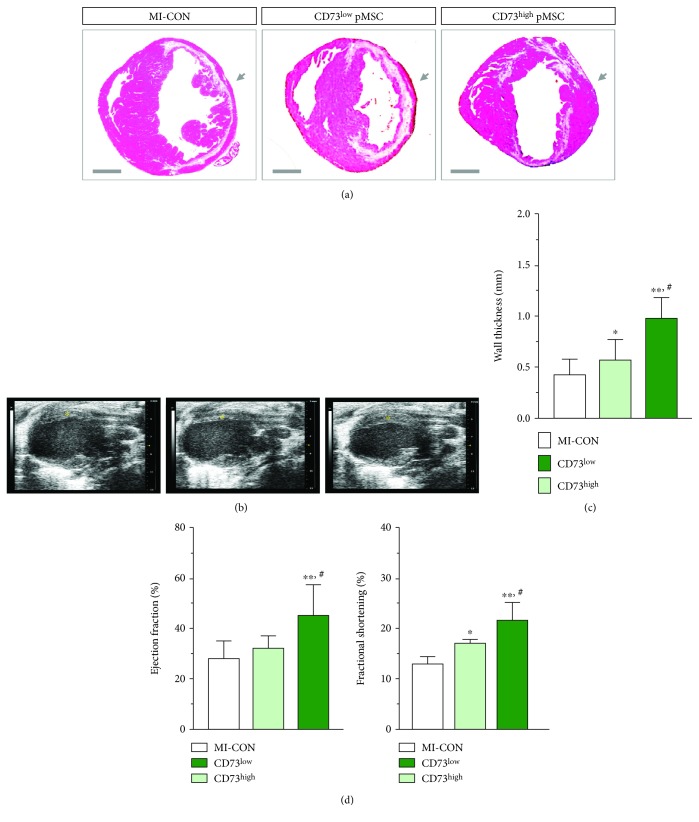
Enhanced cardiac repair by CD73^high^ pMSC in vivo. In comparison to the PBS control (MI-CON, *n* = 5) and CD73^low^ pMSC (*n* = 6), injection of CD73^high^ pMSC (*n* = 5) strikingly reinforced the wall thickness 28 days after transplantation (arrows in (a) and (c)). This result was further confirmed by echocardiography, showing robust thickening of the anterior wall (asterisk in (b)). Furthermore, the structural repair went in parallel with a functional improvement, showing a significant restoration of the ejection fraction and fractional shortening in comparison to the control and CD73^low^ pMSC-treated animals (d). Bar = 1 mm in (a). ^∗^ indicates *p* < 0.05 and ^∗∗^ indicates *p* < 0.01 compared to MI-CON. # indicates *p* < 0.01 compared to CD73^low^ pMSC.

Although CD73 is generally considered as a classical surface marker that defines multipotent mesenchymal stem cells, we found that the CD73 protein was not equally expressed in the freshly isolated pericardial MSC. Flow cytometry revealed that CD73^high^-expressing pMSC constituted a subset of 32.1% of cultivated pMSC at passage 1 (Figure 1(a)). Systematic analysis reveals that heterogenous CD73 expression was also found not only in pMSC but also in MSC from other sources: bone marrow MSC (bMSC) bear 19.2%, subcutaneous adipose MSC (aMSC) 51.8%, and umbilical cord MSC (ucMSC) 84.2% of CD73^high^-expressing MSC (Figure 2(b)), suggesting a ubiquitous phenomenon of uneven CD73 expression within the MSC pool. Immunostaining demonstrated that a distinct subpopulation of pMSC exhibited a strong CD73 expression, while the rest showed a relatively weak expression, differing nearly 5 times in fluorescence intensity (Figure 1(c)). Using a gating strategy in the FACS separation, we subdivided pMSC into two cohorts of the cell population: expression of CD73 at a high level, termed as CD73^high^ pMSC, and expression of CD73 at a low level, termed as CD73^low^ pMSC (Figure 1(c)). The diversity of the CD73 expression was further confirmed by qPCR analysis (Figure 1(d)). Both subsets of pMSC showed a typical cobblestone morphology and proliferated equally under a culture condition with similar population-doubling time (2.3 ± 0.5 vs. 2.4 ± 0.3 days, *n* = 8-9, Figure 1(e)). Furthermore, the two types of pMSC exhibited almost identical surface epitopes (suppl.  Table 1) and differentiation potential towards myogenic, adipogenic, and osteogenic lineages (Figure 1(f)). Therefore, two subpopulations, classified according to the level of CD73 expression, are present in the isolated, cultivated pMSC.

### 3.2. Enhanced Reparative Property in CD73^high^-Expressing pMSC

We further compared the reparative property of the two subpopulations of pMSC in a murine model of myocardial infarction. As demonstrated by the HE and Sirius Red staining in the midventricular section (Figure 2(a) and suppl.  Figure 1), 60 min ischemia caused a massive loss of cardiomyocytes and subsequently collagen deposition, typically resulting in the wall thickness of the left ventricle being as thin as 0.43 ± 0.15 mm in the control animals (MI-CON, *n* = 5, Figure 2(c)). Injection of CD73^low^ pMSC brought about a 20% improvement of the wall thickness 28 days after injection (0.57 ± 0.2 mm, *p* < 0.05, *n* = 6, Figure 2(c)). Injection of the same amount of CD73^high^ pMSC reinforced the anterior wall by doubling the thickness (0.98 ± 0.2 mm, *p* < 0.01, *n* = 5, Figure 2(c)), although both cellular treatments did not alter the infarct size of the hearts (suppl.  Figure 1). Notably, the infarct area was replaced by numerous viable cardiomyocytes (Figure 2(a), red in the HE staining). In this context, the structural repair by injection of CD73^high^ pMSC is apparently more pronounced than by CD73^low^ pMSC, which was further confirmed by echocardiography, showing robust thickening of the anterior wall (indicated by the asterisk in Figure 2(b)). Furthermore, the structural repair by pMSC transplantation was readily translated into a functional improvement after ischemic injury (suppl.  Table 2). The cardiac ejection fraction (EF) and fractional shortening (FS), as indicators of global contractile function, were attenuated in the MI-CON mice (28.1 ± 7.1% and 12.8 ± 1.5%, *n* = 5) and were only slightly improved by treatment with CD73^low^ pMSC (32.3 ± 5.1% and 16.8 ± 0.9%, *n* = 6, *p* < 0.05). Remarkably, mice receiving CD73^high^ pMSC injection demonstrated a significant restoration of EF and FS in comparison to the control and CD73^low^ pMSC-treated animals (45.2 ± 12.2% and 21.4 ± 3.5%, *n* = 5, *p* < 0.01, Figure 2(d)). Therefore, only the CD73^high^ pMSC favors structural and functional benefits that prevent deterioration of cardiac function, underpinning a CD73-dependent beneficial effect in the MI hearts.

### 3.3. Anti-Inflammatory Effects Yielded by CD73^high^ pMSC

**Figure 3:**
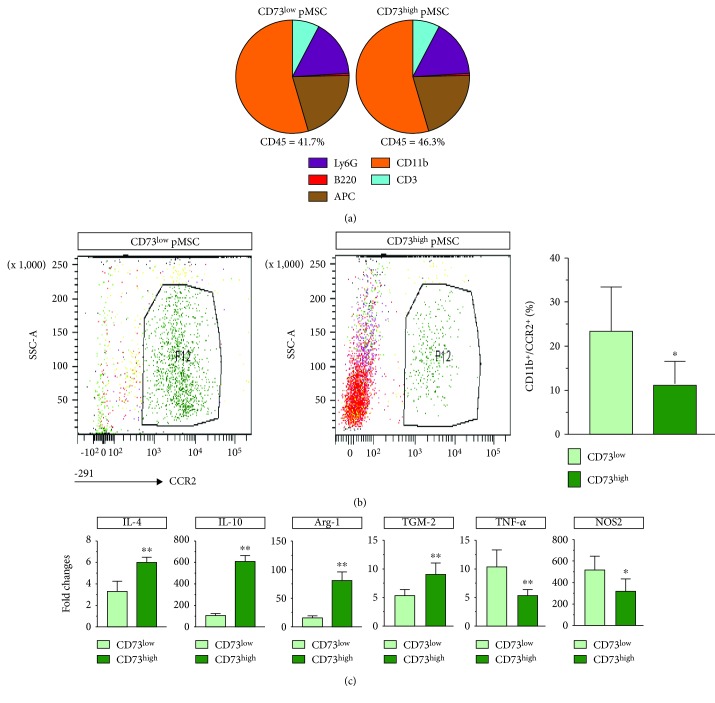
Regulation of the local immune response by CD73^high^ pMSC. The recruitment of immune cells in 5-day MI hearts was not altered by transplantation of either CD73^low^ or CD73^high^ pMSC (a). A subset of macrophages (CCR2^+^) was significantly diminished in amount in the CD73^high^-treated hearts (b). Notably, CD73^high^ pMSC upregulated several anti-inflammatory-related genes (IL-4, IL-10, Arg-1, and TGM-2) and suppressed proinflammatory-related genes (TNF-*α* and NOS2). ^∗^ indicates *p* < 0.05 and ^∗∗^ indicates *p* < 0.01 compared to CD73^low^-treated hearts.

Given that CD73 is able to enzymatically convert AMP into functionally active extracellular adenosine that is able to shape the immune response for cardiac repair [18], we examined the inflammatory status in the hearts 5 days after treatment with pMSC. A total amount of cardiac immune cells (CD45^+^) was almost the same as that in hearts treated with two types of pMSC (41.7%±8.1% vs. 46.3%±11%, *p* > 0.05, Figure 3(a)). Within all infiltrated immune cells, only the number of granulocytes (Ly6G) showed a tendency to increase in the CD73^high^ pMSC-treated hearts (7.7%±1.0% vs 8.9%±0.9%, *p* = 0.065, Figure 3(a)); otherwise, the other population of immune cells including T cells (CD3^+^), B cells (B220^+^), APC (MHC II^+^), and macrophages (CD11b^+^) showed a similar amount in both groups of animals treated with either CD73^low^ or CD73^high^ pMSC, suggesting that immune cell composition was not altered by the treatment with two types of pMSC. Interestingly, we found that the amount of CCR2^+^ macrophages (CD11b^+^/CCR2^+^) was significantly reduced in the CD73^high^ pMSC-treated hearts (*p* < 0.01, Figure 3(b)). Remarkably, the CD73^high^ pMSC-treated hearts demonstrated an enhanced expression of interleukin 4 (IL-4, *p* < 0.01), interleukin 10 (IL-10, *p* < 0.01), arginase-1 (Arg-1, *p* < 0.01), and TGM-2 (*p* < 0.01), while the expression of TNF-*α* and NOS2 was reduced. These results indicate that, although the CD73^high^ pMSC treatment does not impact the percentage of infiltrated immune cells in quantity, it modulates the local immune response by upregulating the expression of several important anti-inflammatory cytokines and by dampening proinflammatory cytokines, which may have paved a proregenerative path towards cardiac repair.

### 3.4. CD73 Modulates the Secretory Profile of Macrophages

**Figure 4:**
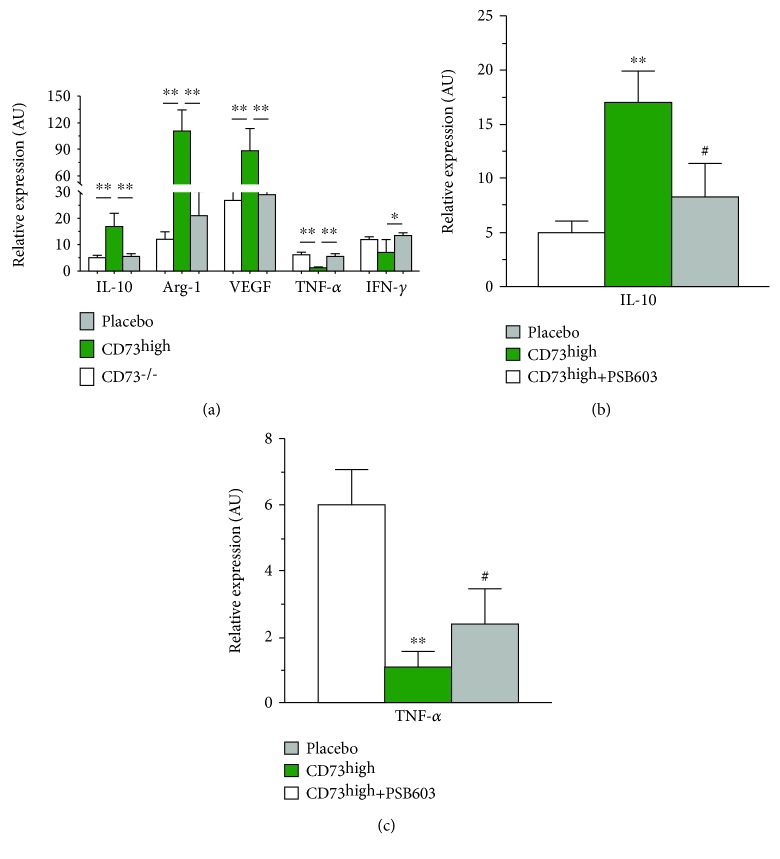
The obligatory role of CD73 and adenosine in the regulation of macrophage activity. When cocultivated with CD73^high^ pMSC, rat peritoneal macrophages (pM*ϕ*) strikingly upregulated several anti-inflammatory cytokines, including IL-10, Arg-1, and VEGF, and downregulated proinflammatory cytokines, including TNF-*α* and IFN-*γ*, in comparison to the placebo control, while pM*ϕ* cocultivated with CD73^−/−^ pMSC did not significantly alter the cytokine profile (a). Pretreatment of pM*ϕ* with the selective antagonist of the A2b receptor of adenosine, PSB603, partially blocked the upregulation of IL-10 induced by CD73 pMSC (b) and restored the inhibitory effects on the TNF-*α* expression (c). ^∗^ indicates *p* < 0.05 and ^∗∗^ indicates *p* < 0.01. # indicates *p* < 0.01 compared to CD73^high^ pMSC.

To analyze the effects of CD73 on immune cells, we conducted in vitro coculture experiments that focus on the interaction of CD73 activity on macrophages. To this end, we also used pMSC isolated from CD73 knockout mice that were genetically lacking the CD73 expression (CD73^−/−^) as a negative control. As shown in Figure 4(a), when cocultivated with CD73^high^ pMSC, peritoneal macrophages (pM*ϕ*) strikingly upregulated several anti-inflammatory cytokines, including IL-10 (threefolds, *p* < 0.01), Arg-1 (ninefolds, *p* < 0.01), and VEGF (3.5-folds, *p* < 0.01), and downregulated proinflammatory cytokines, including TNF-*α* (78%, *p* < 0.01) and IFN-*γ* (58%, *p* = 0.09) in comparison to the placebo control, while pM*ϕ* cocultivated with CD73^−/−^ pMSC did not significantly alter the cytokine profile (Figure 4(a)). The results suggest an obligatory role of the high level of the CD73 expression in generating an anti-inflammatory phenotype of macrophages, a scenario which may also occur likely in an in vivo condition when CD73^high^ pMSC were transplanted into the injured hearts. This notion is supported by pharmacological experiments in which pretreatment of pM*ϕ* with a selective antagonist of the A2b receptor of adenosine, PSB603, partially blocked the upregulation of IL-10 induced by CD73 pMSC (32%, *p* < 0.01, Figure 4(b)) and restored the inhibitory effects on the TNF-*α* expression (27%, *p* < 0.01, Figure 4(c)). Therefore, our in vitro results suggest that the extracellular adenosine, a catalytic product of CD73, serves as an intermediate regulator that masters immune response partially via the activation of the A2b receptor.

## 4. Discussion

The present experiments reveal a nonuniform pattern of CD73 expression in MSC that is associated with the diverse reparative property, although the CD73 molecule is generally considered as a classic surface marker that defines mesenchymal stem cells. Furthermore, our data show that CD73, via its catalytic product of extracellular adenosine, shapes local immune response and is likely to be important in paving a proregenerative road towards cardiac repair.

### 4.1. CD73-Dependent Immunomodulation and Cardiac Repair

Although none of the surface markers specifically identify the MSC population in humans and murines, CD90, CD73, and CD105 are generally accepted as positive identities for the isolation and expansion of MSC [19]. However, it is important to note that differences in cell surface expression of many markers may be influenced by factors secreted by accessory cells in the initial passages, and the in vitro expression of some markers by MSC does not always correlate with their expression patterns in vivo [9]. In this context, we found that the CD73 expression is rather heterogeneous in MSC derived from various sources: with MSC from human umbilical cord blood at the highest and bone marrow-derived MSC at the lowest (Figure 1(b)), suggesting that nonuniform expression of CD73 is a ubiquitous phenomenon in the MSC pool. The heterogeneity of the CD73 expression is not associated with other surface makers that define the MSC population (suppl.  Table 1) or with lineage specification (Figure 1(f)). Given that CD73 expression on immune cells is a pivotal modulator of local immune response after injury [20], we examined the significance of the CD73 level by categorizing pMSC into two cohorts of populations, namely, CD 73^low^ and CD73^high^ pMSC. Our in vivo results demonstrated that the CD73-enriched pMSC harbor more pronounced reparative property in comparison to CD73-weak-expressing cells (CD73^low^ pMSC), which may functionally link to the metabolic capacity of extracellular nucleosides that tightly regulate the local immune response [21]. Therefore, while MSC may use cytokine release, exosomes, and other means to regulate the local immune response, CD73-dependent purinergic signaling is also important to confer reparative property in the damaged hearts (Figure 2).

CD73, also known as ecto-5′-nucleotidase, is an important membrane protein that dephosphorylates extracellular AMP to bioactive adenosine that leads a shift from an ATP-driven proinflammatory environment to an anti-inflammatory milieu [3]. Myocardial ischemia initiates a temporal sequence of the immune response, and uncontrolled excessive inflammation often hampers tissue repair [22]. The finding that delivery of CD73^high^ pMSC yields favorable structural and functional benefits illustrates a crucial role of CD73-mediated adenosine production in orchestrating cardiac inflammation in which several anti-inflammatory genes were upregulated, while proinflammatory genes were suppressed (Figure 3(c)). This result is in line with recent data by Shin et al. who demonstrated that CD73-enriched MSC encapsulated within a hydrogel vehicle increased the bioavailability of extracellular adenosine and reduced cardiac immune response after being implanted onto the damaged myocardium [14]. Therefore, purinergic metabolism may work in conjunction with other described mechanisms such as paracrine factors [6], mitochondrial/organelle transfer, and extracellular vesicle formation [23] to cultivate an anti-inflammatory, proregenerative microenvironment [5], although the relative importance and interaction between these mechanisms remain unresolved [2]. This finding may provide a mechanistic explanation to the inconsistency in published data when using different cell donors which may be related to the intensity of the CD73 expression [8], and thus, the general term of MSC-mediated “immunomodulation” varies upon the expression level of CD73 on MSC from different sources [14].

### 4.2. CD73 Expression and Anti-Inflammatory Activity

Ischemia-induced cardiomyocyte death initiates a local inflammation in three distinct, but overlapping, phases, and individual subsets of immune cells may be implicated correspondingly [3]. Delivery of a high level of CD73 indeed surprisingly did not impact the total amount of immune cells nor the cellular composition of individual cell types that infiltrated into the infarcted heart, which reflects that the recruitment of immune cells to the side of injury is highly dynamic and cannot be overall visualized by a single time point (5 days after MI). Nevertheless, we found that infiltration of a subset of macrophages (CD11b^+^/CCR2^+^) was diminished. CCR2 is the main receptor for the CC chemokine CCL2/MCP-1 and is expressed in Ly-6c^high^ monocytes which display prominent proinflammatory and phagocytotic functions during the first wave of immune cell infiltration [24]. Thus, the presence of CD73^high^ activity yielded an anti-inflammatory milieu by preferentially attenuating the recruitment of macrophages with the proinflammatory phenotype. This scenario was further confirmed by PCR analysis, showing upregulation of anti-inflammatory genes and suppression of proinflammatory genes (Figure 3(c)). Therefore, delivery of CD73 activity tips the balance of the immune response that favors proregenerative activity [20, 25].

Although the anti-inflammatory role of extracellular adenosine on local immune responses has been intensively investigated [18, 25, 26], it is still not clear whether adenosine built by CD73 on MSC functionally regulate the phenotype of macrophages. Thus, we conducted a series of coculture experiments that demonstrated that the presence of CD73^high^ pMSC caused a robust expression of a panel of anti-inflammatory genes and a diminished expression of proinflammatory genes in peritoneal macrophages. Remarkably, the regulatory activity entirely disappeared when cocultivated with pMSC lacking CD73 expression (CD73^−/−^ pMSC), underpinning the obligatory role of CD73 activity to educate macrophages with characteristics resembling a polarized M2 phenotype [27]. Furthermore, we demonstrate that the anti-inflammatory effects were partially mediated by the activation of A2b receptors (Figure 4(b)). Therefore, our in vitro data that mimicked the in vivo situation demonstrated that CD73 activity was obligatorily required to create an anti-inflammatory environment that may pave a proregenerative road to cardiac repair [2]. Although the molecular basis for the actions of macrophages in cardiac repair remains poorly understood [28], the secretion of cytokines and growth factors that modulate fibroblasts and vascular cell phenotypes is likely involved [26].

## 5. Limitations

We are aware of some limitations in the present experiments. First, only the selected panel of the cytokines was tested so far, and in the future, global transcriptional profiling is needed to screen in secretion details in the heart tissue and coculture samples. Secondly, adenosine can stimulate A1, A2A, and A3 receptors and only the A2b receptor antagonist was used in the coculture experiments. Since each receptor has a different affinity to adenosine and activation of distinct receptors may create a profound cellular function, predicting the effect of the individual adenosine receptor activation is complicated particularly in the condition of high CD73 activity. In future study, using macrophages derived from transgenic mice that are lacking expression of individual receptors is desirable.

## 6. Conclusions

Our data reveal that CD73 is unequally expressed in populations of pMSC and that the nonuniform expression leads to a functional diversity: a subset of CD73-enriched pMSC favors cardiac reparative property after transplantation via CD73-mediated regulation of the local immune response. This finding thus provides a possible explanation for the inconsistency of regenerative result when different sources of donor cells were used in cell-based therapy, which may relate to the intensity of the CD73 expression on MSC.

## Data Availability

The raw data and the necessary detail can be provided by the corresponding authors upon reasonable request.

## Supplementary Materials

Supplementary Materials Suppl. Table 1: comparison of surface epitopes in the CD73^low^ and CD73^high^ pMSC. Suppl. Table 2: echocardiographic assessment of cardiac function in mice of the MI control (MI-CON) and treatment with CD73^low^ and CD73^high^ pMSC. ^§^ *p* < 0.05 in comparison to MI-CON; ^∗^ *p* < 0.05 and ^∗∗^ *p* < 0.01in comparison to the CD73^low^ pMSC group. Suppl. Figure 1: comparison of infarct size in the control mice (MI-CON) and treatment with CD73^low^ and CD73^high^ pMSC. Midventricular sections of the representative hearts were stained with Sirius Red for collagen deposition (relevant to myocardial damage) and quantified as a percentage of the total circumference of the left ventricular wall. The results show a similar infarct size in the three groups.
