# Supplementary material for: CD73 Expression on Mesenchymal Stem Cells Dictates the Reparative Properties via Its Anti-Inflammatory Activity
Source: Stem Cells Int. 2019 May 2;2019:8717694. doi: 10.1155/2019/8717694 (PMC6525959; doi:10.1155/2019/8717694)
Supplement: Supplementary Materials — Suppl. Table 1: comparison of surface epitopes in the CD73low and CD73high pMSC. Suppl. Table 2: echocardiographic assessment of cardiac function in mice of the MI control (MI-CON) and treatment with CD73low and CD73high pMSC. § p < 0.05 in comparison to MI-CON; ∗ p < 0.05 and ∗∗ p < 0.01in comparison to the CD73low pMSC group. Suppl. Figure 1: comparison of infarct size in the control mice (MI-CON) and treatment with CD73low and CD73high pMSC. Midventricular sections of the representative hearts were stained with Sirius Red for collagen deposition (relevant to myocardial damage) and quantified as a percentage of the total circumference of the left ventricular wall. The results show a similar infarct size in the three groups. [file 8717694.f1.pdf]

Suppl. Table 1. Comparison of surface epitopes in CD73^{low} and CD73^{high} pMSC.

Markers	CD73^{low}pMSC (n=4)	CD73^{high}pMSC (n=4)
CD 29	99.90±1.2	99.89±0.1
CD 34	1.48±1.2	0.68±0.4
CD 44	99.18±1.3	97.89±1.8
CD 45	0.36±0.2	0.43±0.3
CD 90	99.35±0.7	99.85±0
CD 105	88.85±13.2	90.22±8.5

Suppl. table 2. Echocardiographic assessment of cardiac function in mice of MI control (MI-CON) and treated with $CD73^{low}$ and $CD73^{high}$ pMSC.

	MI-CON (n=6)	$CD73^{low}$pMSC (n=5)	$CD73^{high}$pMSC (n=5)
**IVSd (mm)**	0.29 ± 0.1	0.41 ± 0.1	0.63 ± 0.13**§
**LVIDd (mm)**	4.78 ± 0.8	4.66 ± 0.9	4.72 ± 0.9
**LVPWd (mm)**	0.91 ± 0.1	0.81 ± 0.1	0.86 ± 0.05
**IVSs (mm)**	0.3 ± 0.3	0.48 ± 0.02§	0.74 ± 0.05**§
**LVIDs (mm)**	4.17 ± 0.7	3.88 ± 0.4	3.71 ± 0.6*
**LVPWs (mm)**	1.31 ± 0.2	1.28 ± 0.2	1.18 ± 0.2
**EDV (µl)**	106.7 ± 19.6	100.3 ± 11.4	103.8 ± 13.2
**ESV (µl)**	77.5 ± 9.3	65.5 ± 7.5§	58.4 ± 5.1**§

§= $p<0.05$ in comparison to MI-CON; * = $p<0.05$ and ** = $p<0.01$ to $CD73^{low}$ pMSC group.

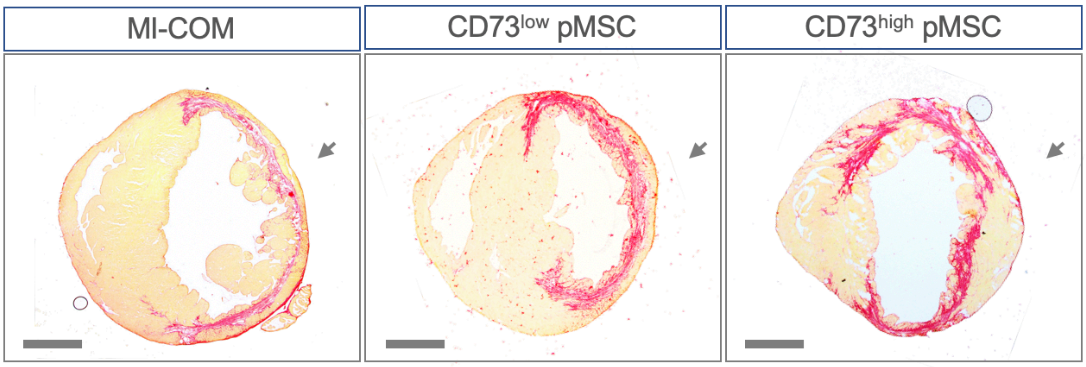

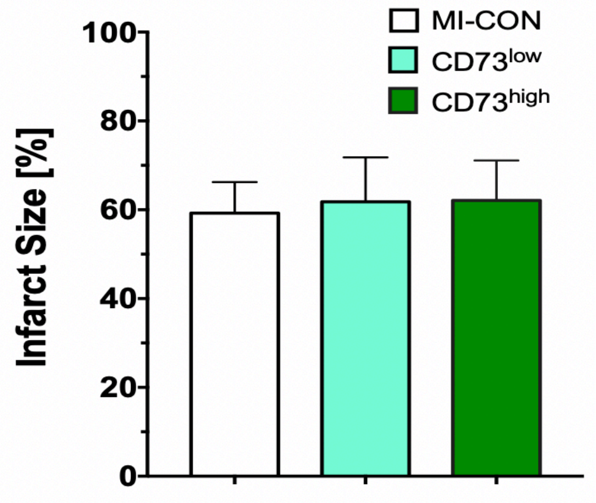

Suppl. figure 1.

Comparison of infarct size in control mice (MI-CON) and treated with CD73^{low} and CD73^{high} pMSC. Mid-ventricular sections of the representative hearts were stained with Sirius red for collagen deposition (relevant to myocardial damage) and quantified as percentage of total circumference of left ventricular wall. The results show a similar infarct size in three groups.
